# Control of Leukocyte Trafficking by Stress-Associated Hormones

**DOI:** 10.3389/fimmu.2018.03143

**Published:** 2019-01-11

**Authors:** Louise M. Ince, Jasmin Weber, Christoph Scheiermann

**Affiliations:** ^1^Department of Pathology and Immunology, Faculty of Medicine, University of Geneva, Geneva, Switzerland; ^2^Walter-Brendel-Centre of Experimental Medicine, University Hospital, Ludwig-Maximilians-University Munich, BioMedical Centre, Planegg-Martinsried, Germany; ^3^DZHK (German Centre for Cardiovascular Research), Partner Site Munich Heart Alliance, Munich, Germany

**Keywords:** catecholamine, glucocorticoid, adrenergic signaling, neutrophil, lymphocyte, circadian rhythm

## Abstract

Leukocyte migration is a crucial process in both homeostatic and inflammatory conditions. The spatiotemporal distribution of immune cells is balanced between processes of cellular mobilization into the bloodstream, their adhesion to vascular beds and trafficking into tissues. Systemic regulation of leukocyte mobility is achieved by different signals including neuronal and hormonal cues, of which the catecholamines and glucocorticoids have been most extensively studied. These hormones are often associated with a stress response, however they regulate immune cell trafficking also in steady state, with effects dependent upon cell type, location, time-of-day, concentration, and duration of signal. Systemic administration of catecholamines, such as the sympathetic neurotransmitters adrenaline and noradrenaline, increases neutrophil numbers in the bloodstream but has different effects on other leukocyte populations. In contrast, local, endogenous sympathetic tone has been shown to be crucial for dynamic daily changes in adhesion molecule expression in the bone marrow and skeletal muscle, acting as a key signal to the endothelium and stromal cells to regulate immune cell trafficking. Conversely, glucocorticoids are often reported as anti-inflammatory, although recent data shows a more complex role, particularly under steady-state conditions. Endogenous changes in circulating glucocorticoid concentration induce redistribution of cells and potentiate inflammatory responses, and in many paradigms glucocorticoid action is strongly influenced by time of day. In this review, we discuss the current knowledge of catecholamine and glucocorticoid regulation of leukocyte migration under homeostatic and stimulated conditions.

## Introduction

**Figure 1:**
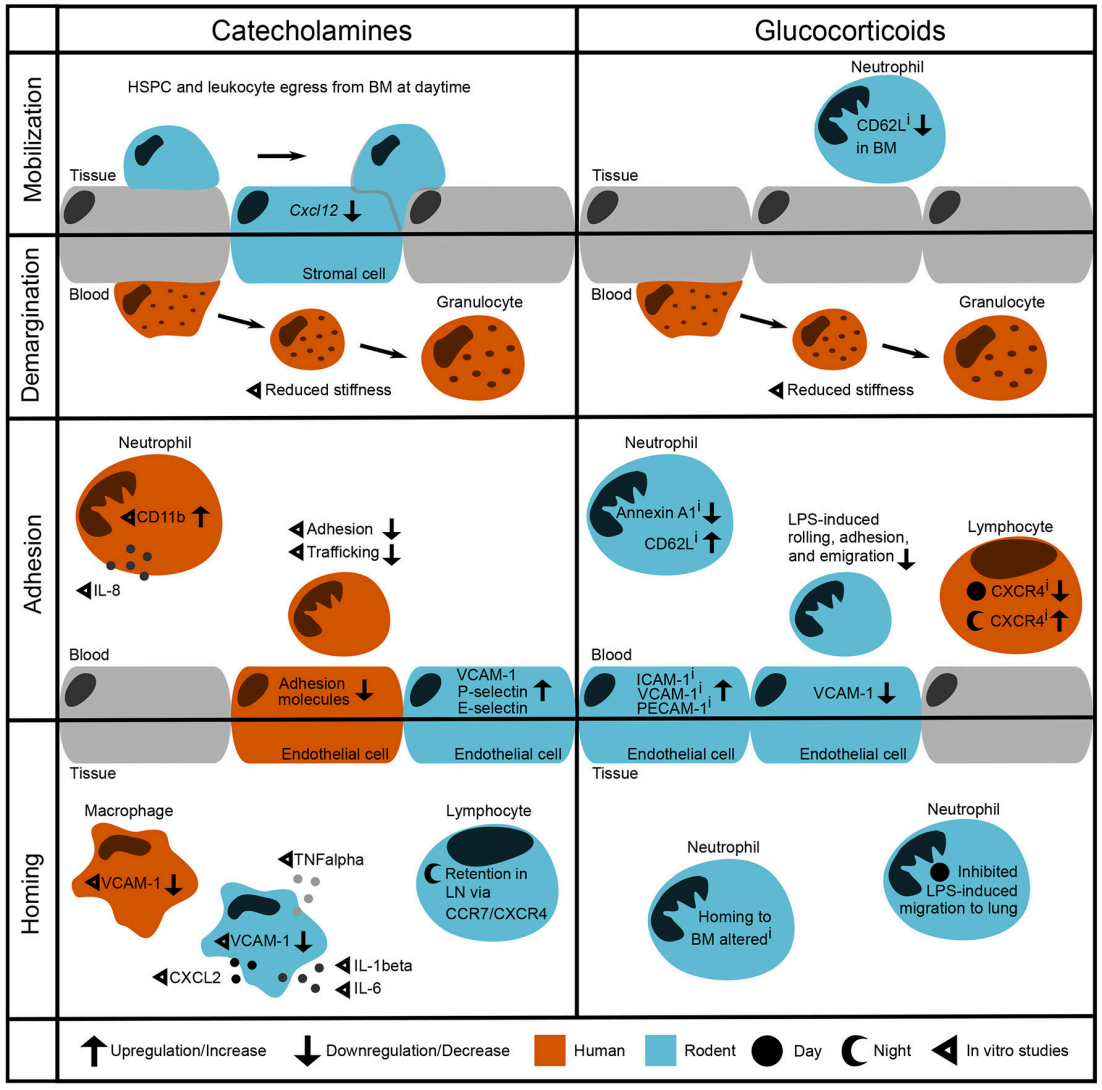
Modulation of leukocyte trafficking by stress-associated hormones. Leukocyte migration can be broadly broken down into mobilization and homing (entering/leaving the vasculature, respectively) as well as adhesion and demargination (attachment to/detachment from the vessel wall, respectively). Catecholamines control hematopoietic stem and progenitor cell (HSPC) and leukocyte egress from the bone marrow during daytime under steady-state conditions by downregulation of the retention factor CXCL12 in stromal cells (2). *in vitro* studies showed that after incubation with catecholamines and glucocorticoids, human granulocytes detach more easily by reducing their stiffness (3). In the bloodstream, human neutrophils show increased levels of CD11b as well as IL-8 after stimulation with adrenaline (4). However, their adhesion and trafficking *in vitro* are reduced due to downregulation of endothelial adhesion molecules (5, 6). In contrast, mouse endothelial cells upregulate VCAM-1, P-selectin, and E-selectin after catecholamine stimulation (7). In both humans and rodent macrophages, VCAM-1 levels are regulated through β_2_-adrenoceptor signaling (8). In addition, catecholamines induce cytokine release by murine macrophages (9). In mice, sympathetic stimulation leads to a retention of T cells in the lymph node via upregulation of CCR7 and CXCR4 (10, 11). Inhibition of glucocorticoid receptors downregulates Annexin A1 levels (12) and upregulates CD62L expression on circulating murine neutrophils whilst downregulating its expression in the bone marrow (13). Furthermore, murine neutrophils show increased LPS-induced adhesion when treated with a GR antagonist—although endothelial VCAM-1 is downregulated (14). Human naïve T cells show upregulated CXCR4 levels when treated with a GR antagonist during the night, whereas CXCR4 is downregulated when treated during the day (15). Similarly, GR agonism with dexamethasone inhibits LPS-induced neutrophil migration to the lung in the behavioral resting phase (16, 17). i denotes effects of inhibition.

Leukocytes migrate through the body by shuttling between the vascular system and tissues. Within the vasculature, immune cells freely circulate or are firmly attached to the vessel wall, effectively removing them from the circulation in what is known as the marginal pool. Adherent cells may be in the process of exiting the circulation to immigrate into organs [reviewed in (1)]. However, still vasculature-bound, marginated cells can also detach and be remobilized into the bloodstream—a process which is called demargination (Figure 1). Leukocytes adhere to the vasculature in a sequence of events known as the leukocyte adhesion cascade. This cascade is crucial for a functioning immune system, allowing immune cells to infiltrate tissues that are in need of pathogen clearance or regeneration. Leukocytes initially roll along the vessel wall with the help of cell adhesion molecules where they can be activated by chemokines on the vascular endothelium, leading to their arrest, and transmigration through the endothelial barrier to exit the bloodstream and enter underlying tissues [reviewed in (18) and illustrated in Figure 1]. These different stages of the adhesion cascade can be modulated by various factors, including circulating hormones such as catecholamines and glucocorticoids. It has been known for decades that the sympathetic nervous system, a key source of catecholamines, regulates the maturation and function of leukocytes via adrenoceptors on their surface [see (19) for an in-depth overview, also on the expression profile of adrenoreceptors]. However, the regulation of leukocyte trafficking by catecholamines and glucocorticoids (typically classed as stress hormones) and their interplay in steady state and stress conditions is multifaceted and therefore incompletely understood. In this review we focus on the recent findings in this field, which we have summarized in Table 1.

**Table 1:** Effects of hormonal signals on leukocyte trafficking.

	**Duration of stimulus**	**Receptor**	**Compound**	**Cell**	**Effect**	**References**
**Catecholamines**	Acute (2 h *in vivo*)	Not assessed	A	Rat CD62L neg. monocytes	Increased numbers in blood	(20)
	Acute (2 h *in vivo*)	Not assessed	A, NA, NA+A	Rat monocytes	Increased numbers in blood, decreased CD62L expression	(20)
	Acute (2 h *in vivo*)	Not assessed	A, NA, NA+A	Rat CD62L neg. neutrophils	Increased numbers in blood	(20)
	Acute (2 h *in vivo*)	Not assessed	A, NA, NA+A	Rat CD62L pos. neutrophils	Increased numbers in blood	(20)
	Acute (2 h *in vivo*)	Not assessed	A	Rat CD62L neg. T, NK cells	Decreased numbers in blood	(20)
	Acute (2 h *in vivo*)	Not assessed	NA+A	Rat CD62L pos T, NK cells	Decreased numbers in blood	(20)
	Acute (2 h *in vivo*)	Not assessed	A, NA, NA+A	Rat NK cells	Decreased CD62L expression	(20)
	Acute (2 h *in vivo*)	Not assessed	A, NA+A	rat lymphocytes	Decreased numbers in blood, decreased CD62L expression	(20)
	Acute (2 h *in vivo*)	Not assessed	A, NA+A	Rat cytotoxic T cells	Decreased numbers in blood	(20)
	Acute (2 h *in vivo*)	Not assessed	NA	Rat CD62L neg. B cells	Decreased numbers in blood	(20)
	Acute (2 h *in vivo*)	Not assessed	A, NA, NA+A	Rat CD62L pos. B cells	Decreased numbers in blood, CD62L expression unaffected	(20)
	Acute (2 h *in vivo*)	Not assessed	NA	Rat B cells	Decreased numbers in blood	(20)
	Acute (2 h *in vivo*)	Not assessed	A, NA+A	Rat B cells	Decreased numbers in blood	(20)
	Acute (4 h *in vitro*)	Not assessed	A	Human neutrophils and monocytes	Increased CD11b expression; suppression of LPS-induced CD11b and CD18 expression	(4)
	Acute (4 h *in vitro*)	not assessed	A	Human white blood cells	Dose-dependent increase in IL-8 levels; suppression of LPS-induced production of IL-1β, IL-8, and CCL2	(4)
	Acute (90 min *in vitro*)	β-AR	A, NA, Isoprenaline	Human PMNs	Reduced fMLP-induced migration, CD11b/CD18 expression and ROS production	(21)
	Acute (30 min pre-treatment *in vitro*)	Not assessed	A	Human neutrophils	Reduced adhesion to HUVECs by down-modulation of EC adhesion molecule expression	(6)
	Acute (30 min *in vitro*)	Not assessed	A, NA	Mouse macrophages/neutrophils	Dose-dependent activation of NFκB, decrease of IκBα levels	(9)
	Acute (4 h *in vitro*)	Not assessed	A, NA	Mouse macrophages	Dose-dependent activation of NFκB, release of TNFα, IL-1β, IL-6, CXCL2	(9)
	Chronic (8 days *in vivo*)	β2-AR	A	Mouse macrophages	Production of IL-6, leading to persistent neutrophil trafficking	(22)
	None (endogenous)	β2-AR	Endogenous	Human/mouse macrophages	Changes in VCAM-1 expression levels	(8)
	None (endogenous)	β2-AR	Endogenous	Mouse lymphocytes	Inhibition of egress from lymph node through CCR7 and CXCR4	(10, 11)
	Acute (20 min *in vitro*)	α2-AR	Xylazine, UK14304	Human neutrophils	Reduced trafficking without affecting CD62L and CD11b expression	(5)
	Acute (6 h *in vitro*)	α2-AR	Xylazine, UK14304	Human endothelial cells	Decreased transendothelial migration of neutrophils	(5)
	Chronic (5 days *in vivo*)	β3-AR	BRL37344	Mouse endothelial cells	Upregulation of VCAM-1, P- and E-selectin expression, more BM homing	(7)
**Glucocorticoids**	Acute (6 h *in vivo*)	GR	Dexamethasone	Human granulocytes	Increased numbers in blood; detached more easily in *ex vivo* assay	(3)
	Acute (2 h *in vitro*)	GR	Dexamethasone	Human granulocytes	Detached more easily in *in vitro* assay	(3)
	Chronic (7 days *in vivo*)	GR	Mifepristone (RU486)^*^	Rat neutrophils	Increased numbers in blood; CD62L expression increased in blood, decreased in BM	(13)
	Int. (24 h and 2 h *in vivo*)	GR	Mifepristone (RU486)^*^	Mouse neutrophils	Decreased annexin A1, altered neutrophil maturation and homing	(12)
	Acute (10 h *in vivo*)	GR	Mifepristone (RU486)^*^	Human T cells	Increased CXCR4 expression in behavioral rest phase, decreased in active phase (inverse to blood numbers)	(15)
	None (endogenous)	GR	Endogenous	Mouse T cells	When T cell GR is disrupted, CXCR4 expression is reduced and homing impaired in active phase	(23)
	Acute (8 h *in vivo*)	MR	Fludrocortisone	Human naïve T cells	Agonism decreased circulating numbers, increased CXCR4 expression (*in vivo*)	(24)
	Acute (2–4 h *in vitro*)	MR	Spironolactone^*^/ Fludrocortisone	Human naïve T cells	Agonism increased CXCR4 and CD62L expression, antagonism decreased CD62L and CCR7 expression	(24)
	Acute (1 h pre-treatment *in vivo*)	GR	Dexamethasone	Mouse leukocytes	Reduced LPS-induced adhesion	(14)
	Int. (18 h and 1 h pre-treatment *in vivo*)	GR	Mifepristone (RU486)^*^	Mouse leukocytes	Increased LPS-induced adhesion, but reduced endothelial VCAM-1 expression	(14)
	Acute (1 h pre-treatment *in vivo*)	GR	Dexamethasone	Mouse neutrophils	Inhibited LPS-induced neutrophil migration into lungs if administered during rest phase, but not during active phase	(16, 17)
	Chronic (trait assessments)	Not assessed	Endogenous	Macaque leukocytes	Positive correlation of cortisol and neutrophil numbers in blood in low-nervous animals, no association in high nervous animals	(25)
	Chronic (16 months) + acute (2 h)	GR	Endogenous (stress) + dexamethasone	Macaque leukocytes	Stressed animals show reduced sensitivity to dexamethasone-induced reduction of circulating lymphocytes	(26)

*Summary of main effects of catecholamines and glucocorticoids upon leukocyte migration as described in the literature*.

* Denotes antagonist;

*A, adrenaline; AR, adrenoceptor; BM, bone marrow; GR, glucocorticoid receptor; MR, mineralocorticoid receptor; NA, noradrenaline*.

## Catecholamines

Catecholamines, such as adrenaline and noradrenaline, are an important class of systemic immune-modulators, released systemically by the adrenal gland and locally mainly by sympathetic nerves. These hormones have immune-enhancing or immune-suppressing effects, depending on the duration of the signal (acute vs. chronic), the microenvironment, and the timing of their release (27). In mice it was demonstrated that under steady-state conditions, the release of hematopoietic stem and progenitor cells requires local delivery of noradrenergic signals to the bone marrow by sympathetic nerves, where they are transmitted to stromal cells via β_3_-adrenoceptors, leading to a downregulation of the key retention factor CXCL12 (2). A similar phenomenon may contribute to the release of leukocytes into the bloodstream in acute stress, as in rats administration of adrenaline and noradrenaline has been shown to increase circulating myeloid and lymphoid cell numbers within a few minutes. In this scenario, most subpopulations have left the blood after 2 h, except for neutrophils, whose numbers continue to increase (20). Differences in the effect on subpopulation specificity are evidenced by the fact that noradrenaline increases numbers of circulating neutrophils and B cells, whereas adrenaline increases the number of neutrophils and monocytes but decreases lymphocyte numbers in blood. (20) (Table 1). The underlying signaling pathways and receptors responsible for these distinct outcomes are, however, ambiguous. For example, it is currently not clear how much of the increase of blood leukocyte numbers is caused by a stress-induced mobilization from hematopoietic tissues into blood, or by demargination from the vessel wall.

Stress hormones can affect leukocyte migratory properties via diverse mechanisms. A recent publication provided the first evidence that catecholamines can induce the rearrangement of cellular cortical actin in human granulocytes, thereby decreasing cell stiffness and leading to leukocyte demargination (3). This could explain the very fast increase in circulating leukocyte numbers by these hormones without the need of mobilization from tissues, allowing the organism to respond quickly to acute signals. Additionally, catecholamines can alter cytokine levels and expression of adhesion molecules. Exposure to adrenaline *in vitro* increases interleukin-8 (IL-8) expression and CD11b (alpha-M-integrin) levels in human neutrophils (4). Under LPS-induced inflammatory conditions the production of IL-1, IL-8, and CCL2 is reduced, indicating that regulation of cytokines and chemokines by adrenaline is highly dependent on the inflammatory milieu (4). In contrast to this study, *in vitro* stimulation with the adrenergic agents adrenaline, noradrenaline, or the agonist isoproterenol reduced N-formyl-methionyl-leucyl-phenylalanine (fMLP)-induced human polymorphonuclear cell (PMN) migration, CD11b/CD18 (Mac-1) integrin expression, as well as production of reactive oxygen species, without affecting IL-8 levels (21). Furthermore, adrenaline and dopamine, a structurally-related catecholaminergic neurotransmitter, facilitated the down-modulation of adhesion molecule expression in human umbilical cord vein endothelial cells (HUVECs), reducing neutrophil adhesion (6). Thus, experiments using catecholamines or their agonists have thus far provided different outcomes, which is most likely dependent on the dosage used and the microenvironmental context. What is clear, however, is that they exert effects on both the immune cell and the endothelial aspects of the adhesion cascade, by modulating expression of adhesion molecules, cytokine levels and leukocyte stiffness.

In addition to their direct influence on the leukocyte adhesion cascade, catecholamines also modulate functions of macrophages, a resident leukocyte subset. As major producers of cytokines, these phagocytic cells are likely largely responsible for the effects of catecholamines on cytokine levels. Adrenaline and noradrenaline can directly activate NF-κB in isolated peritoneal mouse macrophages, resulting in the release of pro-inflammatory cytokines including TNFα, CXCL2, IL-1β, and IL-6 (9). In murine skin wounds, tissue-resident macrophages produce IL-6 in response to chronic β_2_-adrenergic receptor activation, which in turn leads to a persistent trafficking of neutrophils to the site of injury (22). This is one potential mechanism by which long-term stress may be associated with a delayed wound healing. However, phagocytes themselves can also produce catecholamines and in a rat model of acute lung injury, elevated levels of macrophage-derived catecholamines were associated with increased expression of pulmonary intercellular adhesion molecule 1 (ICAM-1) and vascular cell adhesion molecule 1 (VCAM-1) via α_2_-adrenoceptors. Work using knockout models of adrenoceptors could show that in mice and humans the expression of VCAM-1 in macrophages is sensitive to stimulation of β_2_-adrenoceptors, which plays an important role in the cardiac infiltration of leukocytes to facilitate an early inflammatory repair response to an acute myocardial injury (8). Taken together, these findings demonstrate that catecholamines act on resident macrophages but can also be released by these cells, providing an additional, indirect mechanism in regulating the behavior of migratory cells.

β_2_-adrenoceptors are the most common adrenergic receptor type expressed on leukocytes [reviewed in (28)]. However, mRNA for other adrenoceptor subtypes is also present in human immune cells (21). Pharmacological agonists for the α_2_-adrenoceptor reduced trafficking of IL-8 activated human neutrophils by inhibition of CD62L shedding with simultaneous prevention of increased CD11b expression (5). *In vitro* flow chamber assays revealed that targeting the α_2_-adrenoceptor in HUVECs, but not the neutrophils, decreased transendothelial migration of neutrophils (5). These data indicate that both leukocytes and the endothelium are important targets for catecholaminergic signaling in the regulation of leukocyte trafficking. However, the exact mechanisms in different cell types and the interplay of systemic and local factors remain to be identified.

Whereas most studies have investigated the effects of systemic administration of catecholamines and thereby mimicking a stress response, other reports focused on the ablation of catecholaminergic signaling and thus the endogenous role these hormones play in steady state. One study examined the consequence of unilateral surgical ablation of local nerves in mice upon leukocyte adhesion to innervated tissues such as bone marrow and skeletal muscle. Whilst leukocyte adhesion in nerve-intact organs showed a diurnal rhythm (high at night onset, lower during the day), this was abolished in denervated tissues. This pattern corresponded to a rhythmic expression pattern of ICAM-1 in mouse vascular endothelial cells, which was flattened after denervation (7). Rhythms in adherent leukocyte cell numbers were equally lost in mice lacking β_2_- or β_3_-adrenoceptors, indicating that rhythmic adhesion requires local delivery of adrenergic signals by nerves and that the microenvironment is an important regulator of leukocyte trafficking and target site of stress hormones. In murine lymph nodes, activation of β_2_-adrenoceptors leads to the retention of lymphocytes and therefore affects the extent of adaptive immune responses (11). Under steady state conditions, lymphocyte numbers in lymph nodes peak at night (11, 23, 29), which coincides with peak levels of noradrenaline in these tissues (11). After functional depletion of adrenergic nerves using a sympathetic neurotoxin (6-OHDA), restricted lymphocyte egress from the lymph node in the active phase of the animals was observed. The same group had previously demonstrated the physical interaction of β_2_-adrenoceptors with the chemokine receptors CCR7 and CXCR4, which are critically involved in lymphocyte homing to and their retention in murine lymph nodes (10). These data therefore provide evidence for an important time-of-day-dependent regulation of migratory factors on leukocytes and non-hematopoietic cells by β_2_-adrenergic signaling under homeostatic conditions.

Lack-of-function assays are also suited to tease apart the complex interplay of signaling pathways involved in the hormonal regulation of leukocyte migration. Previous data reported that adrenergic signaling through β_3_-adrenoceptors promotes rhythmic egress of hematopoietic stem cells from the mouse bone marrow via downregulation of the retention factor CXCL12 (2). Activation of β_3_-adrenoceptors during the day promotes egress from bone marrow, yet activation of β_2_- or β_3_-adrenoceptors at night promotes homing of murine leukocytes to tissues (2, 7). This apparent paradox was recently investigated in the context of cholinergic signaling, which is a potent inhibitor of endothelial activation in inflammatory scenarios (30) and part of the inflammatory reflex pathway (31, 32). Using mice with decreased cholinergic tone, García-García et al. found that during the day, acetylcholine inhibits vascular adhesion while noradrenergic signals promote egress via β_3_-adrenoceptors, providing complementary effects which increase leukocyte content in blood. At night, the higher circulating adrenaline levels preferentially stimulate β_2_-adrenoceptors while at the same time sympathetic cholinergic signals downregulate β_3_-adrenoceptor expression, promoting nocturnal homing (33). This series of studies highlights the complex interactions between different signaling pathways *in vivo* and the importance of considering neuroendocrine regulation of leukocyte trafficking in an integrative manner.

## Glucocorticoids

The adrenal-derived steroid hormones (glucocorticoids and mineralocorticoids) are another significant class of stress hormones which influence leukocyte migration. Produced in the adrenal cortex, these hormones bind to their cognate receptors [glucocorticoid receptor (GR) and mineralocorticoid receptor (MR)] but with significant overlap. Whilst mineralocorticoids such as aldosterone can only bind MR, endogenous glucocorticoids such as cortisol (humans) and corticosterone (rodents) can bind both receptors. However, due to the higher affinity of endogenous glucocorticoids for MR, this receptor is favored at lower glucocorticoid concentrations and signaling via GR emerges at higher concentrations (34, 35). Appropriate balance between the MR/GR pathways is regulated by the 11β-hydroxysteroid dehydrogenase (11β-HSD) enzymes. 11β-HSD2 converts cortisol and corticosterone into inactive forms, effectively restricting MR signaling to mineralocorticoids in tissues where it is highly expressed, such as the kidney. On the other hand, 11β-HSD1, highly expressed in the liver, can “reactivate” these inactive compounds and locally increase glucocorticoid signaling [see (36) for a review of 11β-HSD functions]. With the DNA-binding domains of human GR and MR showing 94% identity (37), there is also a degree of commonality in their target genes and effects. Innate and adaptive immune cell populations express both MR [reviewed in (38)] and GR, although some sex-specific differences in GR expression levels and isoform distribution are reported in human cells (39). The use of specific, synthetic compounds is therefore more commonly employed to allow more refined investigations into the relative contributions of these pathways, as synthetic glucocorticoids such as dexamethasone show much higher affinity for GR than endogenous ligands (approx. 5-fold) and remain a major class of anti-inflammatory agents in clinical use.

Recently, Fay et al. investigated the influence of glucocorticoid administration on leukocyte demargination and found similar effects to that of catecholamines. Dexamethasone led to increased leukocyte numbers in the bloodstream of patients. *In vitro* experiments showed that dexamethasone increased granulocyte demargination independently of changes in vascular adhesion molecule expression. Although not to the same extent as adrenaline, *in vitro* dexamethasone treatment also induced changes to the actin cytoskeleton, leading to softening of granulocytes and enabling their detachment (3). In addition to effects on biophysical properties of leukocytes, glucocorticoids modulate expression of key receptors on leukocytes to influence maturation, homing, and egress. Neutrophil maturation is accelerated in rats treated with a GR antagonist (mifepristone/RU486) (13), an effect which may be attributable to reduced expression of Annexin A1. Annexin A1 is up-regulated by glucocorticoids, and circulating neutrophils from Annexin A1-deficient mice express higher levels of CXCR4, representing an ‘aged’ phenotype (12). Annexin A1^−/−^ neutrophils did not migrate as efficiently as wild-type cells to CXCL12 *in vitro*, and stromal cells from Annexin A1^−/−^ mice also produced less CXCL12 *in vivo*. The accelerated maturation and inability to home leads to persistent neutrophilia in these mice, and may be a route through which GR antagonism exerts its effects (12). Recent work has also shown this pathway to be involved in the redistribution of T cells (15, 23). In humans, GR antagonism using mifepristone affected T cell CXCR4 expression in a manner dependent on circulating cortisol levels. Using timed administration of mifepristone it was revealed that when endogenous cortisol was low, the GR antagonist increases CXCR4 expression on CD4^+^ and CD8^+^ subsets through a partial agonist effect, whereas administration when cortisol was high led to reduced CXCR4 expression by traditional antagonism (15). This axis has been more extensively investigated in mice, where GR agonism was shown to increase expression of the IL-7 receptor, which then drove increased CXCR4 expression when circulating glucocorticoids were high. Significantly fewer memory CD4^+^ T cells were observed in spleen, lymph node, and lungs of mice lacking GR in T cells than in wild type controls, suggesting that cell-intrinsic GR signaling enhances survival of this population and promotes migration to peripheral lymphoid tissues (23). Furthermore, MR signaling also increases CXCR4 expression on naïve human T cells but does so along with CD62L and CCR7, suggesting that MR activation facilitates homing to lymph nodes whereas GR activation preferentially drives cells toward the bone marrow (24). In an inflammatory scenario, glucocorticoid administration generally inhibits the immune response, as GR activation decreases expression of many pro-inflammatory cytokines. In a mouse model of LPS-induced inflammation, dexamethasone treatment also resulted in reduced leukocyte rolling flux, adhesion and emigration, along with reduced circulating leukocyte counts, whereas mifepristone treatment increased adhesion and emigration (14). These data show that GR agonism attenuates interactions between leukocytes and the endothelium in this model, consistent with dexamethasone-induced inhibition of ICAM-1 and VCAM-1 expression on the inflamed endothelium. Interestingly, the blockade of endogenous GR signaling by mifepristone resulted in a counter-intuitive decrease in VCAM-1 expression, suggesting that there may be a difference between endogenous and exogenous glucocorticoids and their effects on leukocyte-endothelium interactions (14). It will be interesting to see whether further studies can dissect the relative contributions of endogenous or exogenous glucocorticoids and their signaling through GR and/or MR.

In addition to sensitivity of adrenergic and glucocorticoid signaling to acute environmental signals and stressors, these signals are also regulated on a longer time scale by the circadian rhythm [see (40) for a review of circadian regulation of immune function]. Circulating glucocorticoids and adrenergic tone both increase at the start of an organism's behavioral active phase, providing a rhythmic signal promoting redistribution of leukocytes across the body. The influence of such rhythmic signal is seen in the results of Besedovsky et al. (15), where the diurnal oscillation in endogenous cortisol significantly influenced the ability of GR antagonism to elicit changes in human T cell CXCR4 expression. Shimba et al. (23) also addressed the role of GR in a rhythmic manner, supporting data by other groups (11, 29) and providing an additional mechanism to regulate leukocyte trafficking in a daily cycle. In inflammatory scenarios a rhythmic glucocorticoid signal is known to modulate chemokine signaling and neutrophil trafficking to the mouse lung via time-of-day dependent inhibition of epithelial CXCL5 production (16, 17), providing an additional layer of fine-tuning the inflammatory response. Under chronic stress conditions, however, elevated glucocorticoid levels are associated with a reduction in cellular sensitivity to these hormones. Experiments using rhesus macaques have illustrated a link between both nervous temperament and social stress and impaired leukocyte trafficking patterns (25, 26). Whilst control animals showed glucocorticoid-induced redistribution of circulating leukocytes, those exposed to social stress showed a reduced correlation between cortisol concentration and blood lymphocyte content (26). In further experiments without social manipulation but with analysis of behavior and temperament, the expected correlation between cortisol and blood neutrophil counts was found at a population level, but this was significantly attenuated in nervous macaques (25). These results have interesting implications for human scenarios of disrupted neuroendocrine functions, stress, and anxiety. In these situations, a disconnection appears between circulating hormone levels and inflammatory cell responsiveness, which may explain the lack of efficacy of glucocorticoid treatment in some patients. Furthermore, there may even be a cycle of inflammatory exacerbation due to the effects of stress upon monocyte trafficking and microglial activation, whereby reactive endothelium and enhanced trafficking of cells to the brain releases cytokines and reinforces stress- and anxiety-like signaling [reviewed in (41, 42)]. Similar to catecholamines, glucocorticoids are key systemic orchestrators of immune cell migration. Yet, due to the complexity of the interlocking signaling cascades in different leukocyte subsets and tissues, the precise effects in different sites of the body remain elusive.

## Conclusion

In summary, the hormones adrenaline, noradrenaline and glucocorticoids, typically associated with a stress response, exert diverse effects on leukocyte migration under both steady-state and stimulated conditions. These effects are dependent not only on the responding cell type but also on location, duration and source of the stress/hormone signal, inflammatory context, and even time of day. Whereas adrenaline increases circulating neutrophil numbers, it reduces lymphocyte numbers in blood. Noradrenaline, on the other hand, increases both neutrophil and B cell numbers with distinct temporal profiles. Glucocorticoids can act to redistribute T cells from the bloodstream into organs at their endogenous peak levels, but synthetic agonists are widely used in inflammatory scenarios to inhibit chemokine production and disrupt excessive inflammatory responses. This potential divergence between the function of endogenous hormones and their clinical counterparts should be explored further, particularly with respect to cell-specific differences in receptor expression and diurnal rhythms in endogenous hormone concentrations. To achieve this, analyses using lineage specific ablation of hormone receptors will be needed in combination with well-controlled *in vitro* and *in vivo* studies to dissect their complex and highly interwoven signaling pathways and functions.

## Author Contributions

All authors listed have made a substantial, direct and intellectual contribution to the work, and approved it for publication.

### Conflict of Interest Statement

The authors declare that the research was conducted in the absence of any commercial or financial relationships that could be construed as a potential conflict of interest.
